# Characterisation of the SNAP-25 and SNAP-23 cleavage properties of the botulinum neurotoxin-like protein from Enterococcus

**DOI:** 10.1007/s00702-025-03082-z

**Published:** 2025-12-22

**Authors:** Tina Henke, Gema Morillas Ramos, Laurien Czichon, Thomas Binz

**Affiliations:** Institut für Zellbiochemie, OE 4310, Medizinische Hochschule Hannover, 30623 Hannover, Germany

**Keywords:** SNAP-25, SNAP-23, Zinc protease, Botulinum neurotoxin, Neurotoxin engineering, Substrate specificity

## Abstract

Botulinum neurotoxins (BoNT) are well-established biopharmaceuticals used to treat neuromuscular, secretory and pain conditions. They do this by blocking neurotransmitter release from neurons through the proteolysis of specific soluble N-ethylmaleimide-sensitive-factor attachment protein receptor (SNARE) proteins. Current research seeks to expand the range of BoNTs applications to other medical conditions. One possible starting point for this endeavour is the catalytic domain of the BoNT-like protein form *Enterococcus faecium*. This protein has been reported to cleave the BoNT resistant human SNAP-23 (hSNAP-23) isoform of SNAP-25 (synaptosomal associated protein of 25 kDa), which is the substrate of the primary BoNT pharmaceutical, BoNT serotype A1. SNAP-23 is an attractive new target because it has been reported to be involved in the excessive release of inflammatory messengers. Therefore, we characterized the substrate cleaving properties of the catalytic domain of this BoNT-like protein. Biochemical characterization showed that LC/En cleaves hSNAP-23 approximately 25 times less efficiently than SNAP-25. However, compared to LC/A, LC/En readily cleaves hSNAP-23, excelling the activity of LC/A by more than 100-fold. To lay the groundwork for later re-engineering of LC/En to tailor its enzymatic activity to hSNAP-23, we characterized the enzyme–substrate interaction through systematic mutational analysis of hSNAP-23. We found that the amino acids in the P2 and P1' positions are crucial and that the substitutions at amino acid positions 49 and 53 versus SNAP-25 largely explain hSNAP-23’s 25-fold lower cleavage rate compared to SNAP-25. Therefore, the binding pockets of hSNAP-23 Lys-49 and Asn-53 in LC/En are prime targets for adapting the enzyme to increase its cleavage of hSNAP-23.

**Supplementary Information:**

The online version contains supplementary material available at 10.1007/s00702-025-03082-z.

## Introduction

Various species of the bacterial genus Clostridium synthesise botulinum neurotoxins (BoNTs). These protein toxins are considered the most hazardous natural substances to humans. Yet, for over 30 years, serotype A (BoNT/A) and, for certain conditions, serotype B (BoNT/B) have been used as effective pharmaceuticals to treat of medical conditions caused by hyperactivity of cholinergic nerve terminals. Since BoNT/A was first approved to treat blepharospasm, hemifacial spasm, and strabismus, its range of applications has steadily expanded to include various neuromuscular and autonomic disorders, as well as applications in aesthetic medicine (Jankovic 2017; Pirazzini et al. 2017, 2022).

In addition to the seven classical BoNT serotypes (A-G) and various BoNT subtypes within serotypes A, B, E, and F, recently another serotype (type X) and several non-clostridial BoNT-like proteins have recently been discovered (Peck et al. 2017; Dong et al. 2019). Botulinum neurotoxins are synthesized as ~ 150 kDa single-chain proteins that exhibit three domains. An enzymatic light chain (LC) of ~ 50 kDa at the N-terminus is followed by a ~ 50 kDa translocation domain (H_N_), which comprises the N-terminal half of the heavy chain (HC). Subsequent to receptor mediated uptake, this domain forms a channel in endocytic vesicles triggered by acidification and delivers the LC to the cytosol of the target cell. The C-terminal half of the HC (the ~ 50 kDa H_C_ domain) ensures the highly specific binding to neuronal cells via polysialogangliosides and endocytosis via proteins of recycling synaptic vesicles. Once in the cytosol, the LCs act as zinc metalloproteases, hydrolysing a single peptide bond in specific SNAREs (soluble N-ethylmaleimide-sensitive factor attachment protein receptors). BoNT/B, D, F, G, and X cleave synaptobrevin/vesicle-associated membrane protein (VAMP) isoforms 1, 2, and 3. BoNT/A, and BoNT/E cleave SNAP-25 (synaptosomal-associated protein of 25 kDa), while type C attacks SNAP-25 and syntaxins 1, 2, and 3. The cleavage of these SNAREs blocks neurotransmitter release for a serotype-specific duration, causing the characteristic botulism symptom of a flaccid paralysis. This can lead to respiratory failure and death (Pirazzini et al. 2017, 2022). BoNT/X is the only toxin that cleaves the non-canonical substrates VAMP4, VAMP5 and Ykt6 in addition to the aforementioned substrates (Zhang et al. 2017).

Current research aims to expand the use of BoNTs to treat additional medical conditions by altering the specificity of a particular LC. For example, this could involve refractory SNAREs that are involved in secretion from non-neuronal cells, as hypersecretion from these non-neuronal cells may be the cause of widespread diseases such as chronic obstructive pulmonary disease, asthma, and diabetes. Besides refractory SNAREs, another obstacle is the lack of appropriate receptor molecules on the target cells. However, reports in the literature suggest that the issue of delivering BoNT LCs to cells not naturally targeted by the toxins can be achieved using technologies such as the targeted secretion inhibitor (TSI) platform or SNARE tagging technology (Darios et al. 2010; Fonfria et al. 2018). These approaches combine an engineered LHN (LC and HN translocation domain) with a binding domain that allows binding to a cell surface receptor and subsequent endocytosis at the desired target cell.

LCs tailored for refractory SNAREs that might play a role in disease-causing hypersecretion in non-neuronal tissue have also been developed. Screening methods combined with rational design have yielded an LC/A mutant that readily cleaves human SNAP-23 (hSNAP-23) (Sikorra et al. 2020; Dyer et al. 2022). hSNAP-23, an isoform of SNAP-25, is involved in secretion from non-neuronal cells, such as myeloid cells, and is not hydrolysed by the prototypical BoNT/A1 or any of the growing number of characterised BoNT/A subtypes (Henkel et al. 2009; Wang et al. 2013; Kull et al. 2015). Furthermore, Blum and colleagues utilised an M13 phage-assisted evolution system, which allows the application of both positive and negative selection constrains to redirect the protease specificity (Blum et al. 2021). Using this method, LC/X was evolved into variants that cleave the VAMP family members VAMP4 and Ykt6 preferentially, and LC/F was enabled to cleave VAMP7 (Blum et al. 2021). This approach even enabled the generation of an LC/E derivative that cleaves a non-SNARE protein, namely phosphatase and tensin homolog (PTEN), but no longer its authentic substrate SNAP-25 (Blum et al. 2021).

In 2018, a BoNT-like protein (BoNT/En) encoded in the Enterococcus faecium genome was identified and characterized (Brunt et al. 2018; Zhang et al. 2018). Its LC (LC/En) exhibits a wide-ranging activity towards SNAREs. In in vitro cleavage assays, it proteolyses VAMP-1, -2, and -3 as well as syntaxin (Syx)-1B and Syx-4, the latter two only at a high LC concentration (Zhang et al. 2018). Furthermore, SNAP-25 and hSNAP-23, which resists BoNT, proved to be substrates of LC/En. Notably, compared to BoNTs, LC/En hydrolyses members of all three SNARE families, each one at a unique peptide bond. Additionally, unlike SNAP-25-cleaving BoNTs, the scissile peptide bond of LC/En in SNAP-25B, the Lys69-Asp70 bond (Lys64-Asp65 in hSNAP-23), resides in the first SNARE domain rather than the second SNARE domain located at the C-terminus (Zhang et al. 2018).

Here, we characterized the proteolytic properties of the LC of this BoNT-like protein to determine whether it exhibits superior hSNAP-23 cleavage features to LC/A and engineered LC/A. Our data indicate that LC/En and LC/A have a similar catalytic mechanism. We demonstrate that the efficiency of SNAP-25 cleavage by LC/En is approximately 10 times lower than that of LC/A. However, LC/En surpasses LC/A by approximately 100-fold in terms of hSNAP-23 cleavage. Additionally, we demonstrate that the reduced efficiency of hSNAP-23 relative to SNAP-25B cleavage is primarily due to the replacements of glycine to lysine and asparagine to glutamic acid in positions 49 and 53 of hSNAP-23, which are located 12 and 16 amino acids upstream of the scissile peptide bond. This finding provides a starting point for tailoring LC/En through targeted mutations to increase its activity with respect to hSNAP-23. Based on its intrinsic proteolytic activity against hSNAP-23, such an engineered LC/En should be used as a building block in the development of novel pharmaceuticals for treating diseases that rely on SNAP-23-mediated hypersecretion.

## Materials and methods

### Plasmid constructions

The cDNA for full-length human SNAP-23 (Galli et al. 1998) was cloned in a modified pRSET5a (accession X54202) vector, yielding pS3-hSNAP-23His6. First, the One-STrEP encoding segment of pIBA105-strep (accession: MT966996) was inserted via NheI and EcoRI. Secondly, the hSNAP-23 cDNA carrying the coding capacity for GHHHHHH at the 3'-end was inserted by using the NarI and HindIII sites. The plasmids used for synthesis of mouse SNAP-25A (accession: NP_001342183.1) and SNAP-25B (accession: NP_001277985.1) by in vitro transcription/translation were assembled in pSP73 (Promega, Walldorf, Germany) using the BamHI and SalI sites. Both plasmids carry the coding capacity for eight additional amino acids (SGHHHHHH) following the C-terminal Gly-206. N-terminal deletion mutants of mouse SNAP-25A were generated by PCR and cloned in pQE3 using the BamHI and PstI sites. Each mutant encodes the C-terminal SGHHHHHH-affinity tag. C-terminal deletion mutants of mouse SNAP-25B were cloned in pET41 (Merck Millipore, Darmstadt, Germany) using the NcoI-and HindIII sites giving rise to N-terminally GST- and C-terminally His8-tagged proteins.

A bacterial expression plasmid for the LC (amino acids 1 to 433) of the botulinum neurotoxin-like protein from Enterococcus was generated by inserting a synthetic DNA (optimized for E. coli) in pQE9 using the BamHI and HindIII sites, yielding pQE9-LC-En.

### Production and purification of recombinant proteins

LC/En as well as the N-terminal truncation mutants of SNAP-25A were produced in the E. coli strain M15[pREP4] (Qiagen, Hilden, Germany) during 15 h of induction at 21 °C in the presence of 0.25 mM IPTG. LC/En and the N-terminal deletion mutants of SNAP-25 were purified on Ni2 + -nitrilotriacetic acid-agarose (NTA) beads, eluted with 100 mM imidazole and dialyzed against 10 mM HEPES/KOH, pH 7.2, supplemented with 150 mM potassium glutamate (toxin assay buffer), shock-frozen in liquid nitrogen, and stored at -70 °C until use. C-terminally truncated mutants of SNAP-25B were produced in the E. coli strain BL21-DE3 and purified on Glutathione sepharose 4B beads (GE Healthcare, Munich, Germany), eluted with 10 mM glutathione, and in the final step dialyzed against toxin assay buffer. Protein concentrations were determined following thawing of frozen aliquots, SDS-PAGE analysis and Coomassie blue staining by means of the LAS-3000 imaging system (Fuji Photo Film, Co., Ltd.) and the Multi Gauge software (Fuji Photo Film). Various known concentrations of bovine serum albumin were run as standard. Full-length SNAP-25A, SNAP-25B and hSNAP-23 were generated by in vitro transcription/translation via the above described plasmids, using either the SP6 or T7 coupled TNT reticulocyte lysate system (Promega, Walldorf, Germany), and L-[35S]methionine (370 kBq/µL, > 37TBq/mmol, Hartmann Analytic, Braunschweig, Germany) according to the manufacturer's instructions.

### Endopeptidase assays

**Fig. 1:**
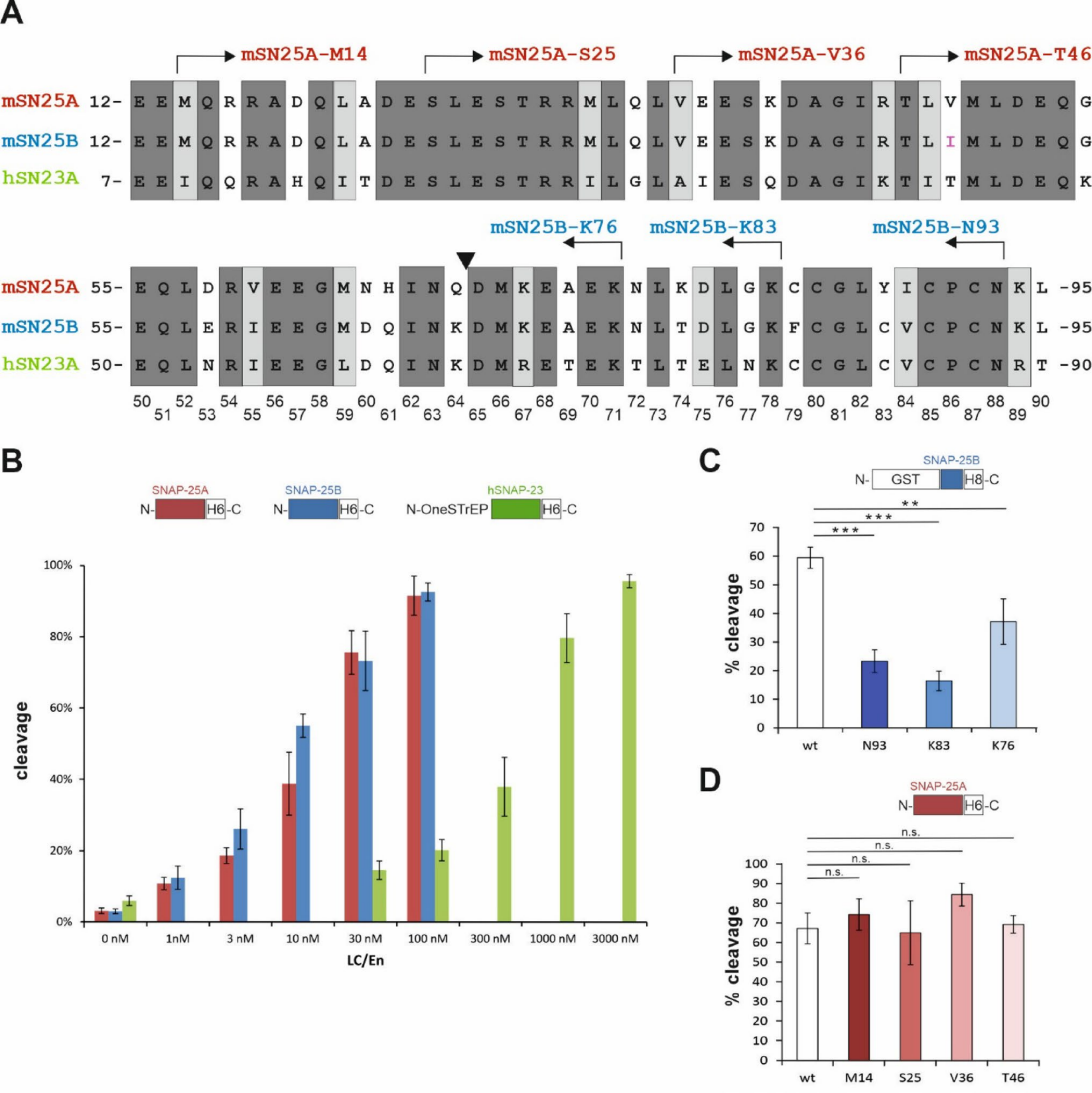
Cleavability of SNAP-25A, SNAP-25B and hSNAP-23 by LC/En. **A** Amino acid alignment of SNAP-25A, SNAP-25B and hSNAP-23. Conserved amino acids and those with similar characteristics are shown on dark grey and light grey background, respectively. Arrows above the alignment specify the boundaries of N- and C-terminally truncated SNAP-25 variants used for cleavage analyses. The closed triangle marks the scissile peptide bond for LC/En. The numbers below the alignment denote hSNAP-23 amino acid positions. **B** Cleavability of full-length SNAP-25A, SNAP-25B and hSNAP-23. Each substrate was generated by in vitro transcription/translation as 35S-labled, His6-tagged protein and incubated in the presence of various concentrations of LC/En for 1 h at 37 °C. Reactions were stopped by the addition of pre-chilled SDS-PAGE sample buffer. Samples were boiled, and then run on 15% gels. After fixation of proteins, gels were dried and radioactively labelled proteins visualized by phosphorimaging. The values shown in the bar diagram represent the mean ± SD of four to six independent experiments. **C, D** Cleavage analyses of C-terminally (**C**) and N-terminally (**D**) truncated SNAP-25 variants. Truncated SNAP-25 variants were produced in E. coli and purified via their affinity tags and incubated in the presence of a 30 nM final concentration of LC/En for 1 h at 37 °C. After addition of sample buffer and boiling, the samples were run on 15% gels and proteins detected by Coomassie brilliant blue staining. The values shown represent the mean ± SD of four independent experiments. A one-way ANOVA followed by Bonferroni post-hoc tests was as used to calculate the statistical significance (*** p ≤ 0.001, ** p ≤ 0.01)

**Fig. 2:**
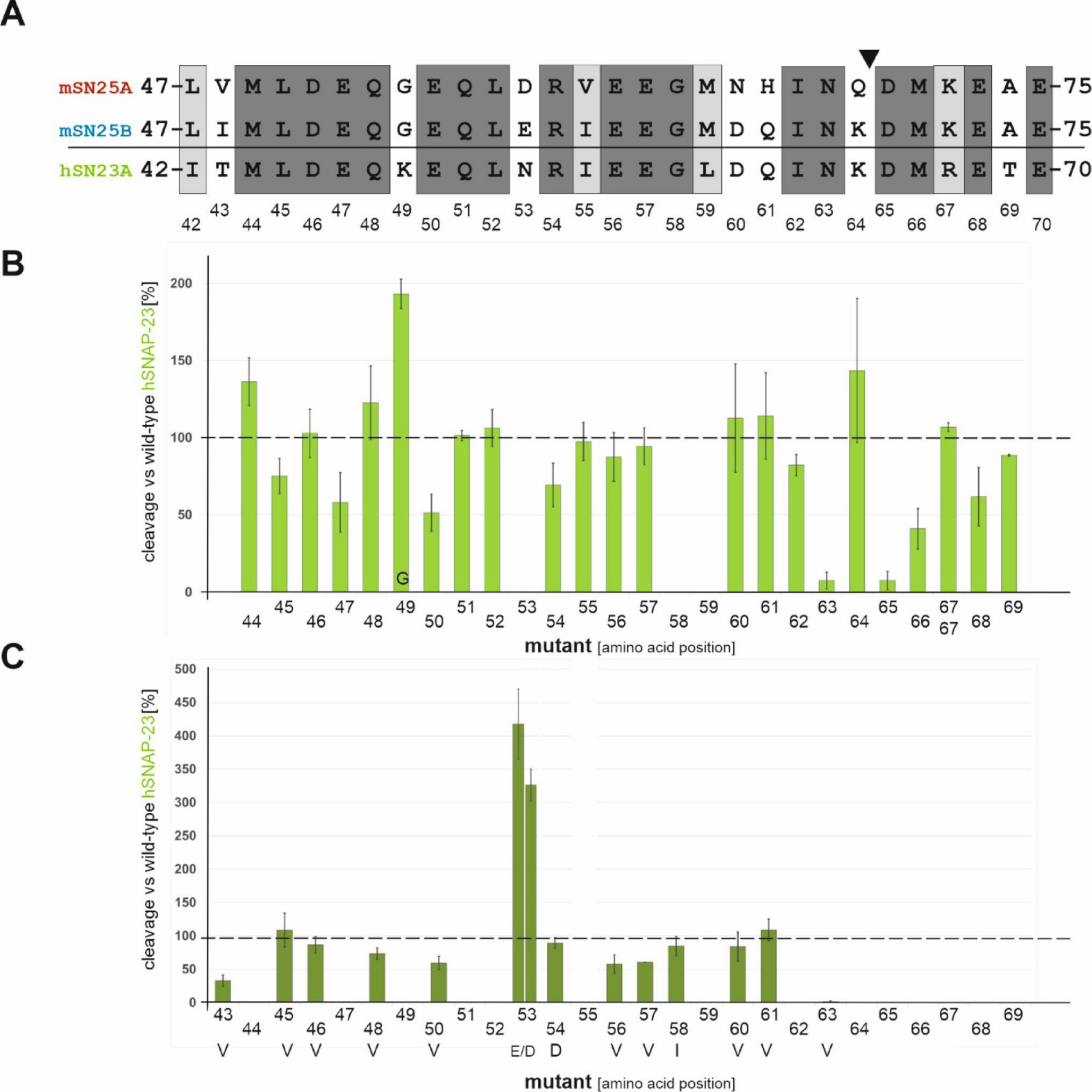
Cleavability of hSNAP-23 mutants. (A) Amino acid alignment of SNAP-25A, SNAP-25B and hSNAP-23. The numbers below the alignment denote hSNAP-23 amino acid positions. (**B, C**) Analysis of mutations in the hSNAP-23 area of the previously determined minimal substrate for LC/En. All mutants were generated by in vitro transcription/translation as ^35^S-labelled proteins and incubated in the presence of LC/En for 1 h at 37 °C. After stopping the reactions, the samples were subjected to SDS-PAGE (15% gels). After fixation of proteins, gels were dried and radioactively labelled proteins visualized by phosphorimaging. Finally, the extent of cleavage was calculated using the Multi Gauge software. The bar diagrams in (**B**) show data of an alanine scanning approach (except that Lys-49 was replaced with glycine). The bar diagram in (**C**) depicts data for mutations of hSNAP-23 residues to the bulky side group carrying valine or an acidic amino acid. All data are expressed as %-cleavage versus wild-type hSNAP-23, calculated for concentrations at which the extent of cleavage did not exceed 70%, i.e. N53D and N53E at 30 nM, K49G and K64A at 100 nM, and all remaining mutants at 300 nM final concentration. The values represent means ± SD of three to five independent experiments

**Fig. 3:**
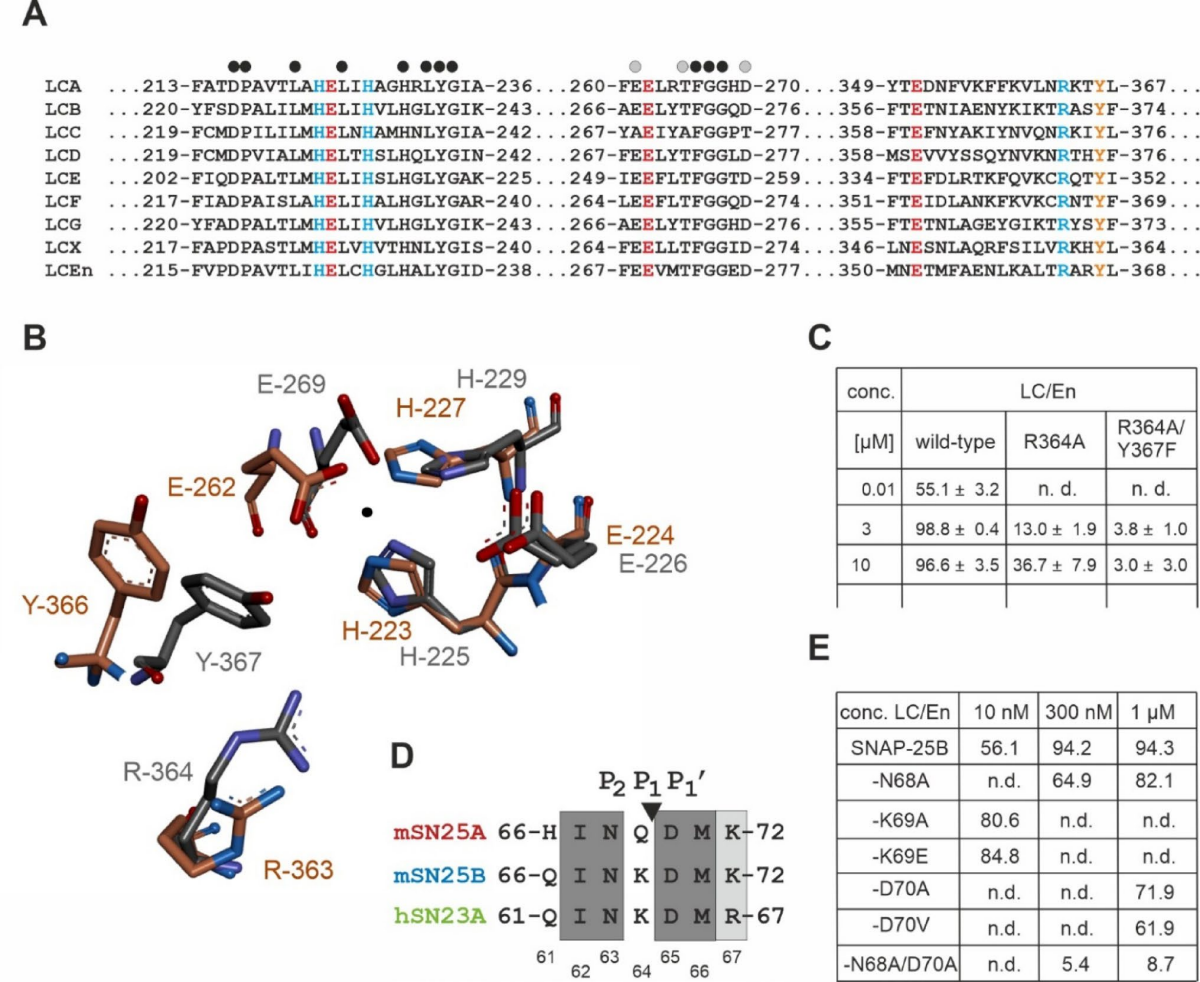
Role of Arg- and Tyr- of the active centre of LC/En in the catalytic mechanism. **A** Alignment of BoNT LCs and LC/En amino acids sections comprising the residues involved in the catalytic mechanism. Residues attributed to a function in the catalytic mechanism are coloured. Strictly conserved amino acids are marked by a black dot, while positions conserved in all but one protein are marked by a grey dot. **B** Close-up view of active centre amino acids of LC/En depicted in stick representation in comparison with those of LC/A (PDB ID: 1XTG). LC/En carbon atoms are shown in grey, whereas LC/A carbon atoms are shown in orange. The catalytic zinc ion is presented as grey sphere. **C** Proteolytic activity of the LC/En mutants R364A and 364A/Y367F on SNAP-25B. Values depicted are means of three experiments ± SD. **D** Alignment of SNAP-25 and SNAP-23 amino acids of the P_4_ to P_3_' region. Conserved amino acids and those with similar characteristics are shown on dark grey and light grey background, respectively. The numbers below the alignment denote hSNAP-23 amino acid positions. **E** Cleavability of SNAP-25B mutants of the P2, P1 and P1' positions. Values depicted represent means of two experiments

Cleavage assays using SNAP-25 produced in E. coli were conducted in a total volume of 20µL exhibiting a 10 µM substrate concentration and a final LC/En concentration as specified in figure captions. Cleavage assays employing radiolabelled substrate contained 2µL of the transcription/translation mixture and 18µL of LC/En in toxin assay buffer at various final concentrations as specified in Fig. 3 and the captions to Figs. 1, 2. Protease reactions were run for 60 min at 37 °C and stopped by the addition of 20µL of double-concentrated sample buffer [120 mM Tris–HCl, pH 6.75, 10% (v/v) β-mercaptoethanol, 4% (w/v) SDS, 20% (w/v) glycerol, 0.014% (w/v) bromophenol blue]. Samples were incubated at 99 °C for 2 min and then subjected to SDS-PAGE using 12.5% or 15% gels. Gels containing substrates produced in E. coli were stained with Coomassie blue and scanned with the LAS-3000 imaging system. Gels used for analysing cleavage of radiolabeled substrates were dried and radiolabelled proteins were visualised employing a FLA-9000 phosphorimager (Fuji Photo Film, Co., Ltd., Tokyo, Japan). Quantification of proteins and their cleavage products was done using the Multi Gauge software (Fuji Photo Film). The percentage of cleavage was calculated by dividing the sum of the signal intensities of bands 3, 4, and 5 (after subtracting the corresponding signals for the untreated sample (0 µM), indicated by red rectangles) by the sum of the intensities of bands 1 to 5, also indicated by red rectangles) and then multiplying by 100 (Fig. S1).

### Protein structure analyses

A structural model for LC/En was generated in 2018 using the SWISS-MODEL homology modelling tool (Biasini et al. 2014) before the X-ray structure data of LC/En (pdb code: 8OW8; (Gregory et al. 2023)) became available. The modelled structure and the experimentally solved structure did not show any relevant deviations. Structure analyses and the manually aligned superposition of the modelled structure with SNAP-25 bound LC/A (pdb code: 1XTG; (Breidenbach and Brunger 2004)) and image preparation were done using the Discovery Studio Visualizer v4.5.0.15071 software (Dassault Systemes BIOVIA, Munich, Germany).

**Statistical analyses.** For statistical analysis IBM SPSS Statistics v. 30.0.0.0 was used (Chicago, IL, USA). A one-way ANOVA followed by Bonferroni post-hoc tests was used to compare the means of the different groups. Significance levels were set at 0.05 for all analyses.

## Results

### Sensitivity of SNAP-25 and SNAP-23A to proteolysis by LC/En

BoNTs that target SNAP-25 are virtually inactive toward the SNAP-25 family member human SNAP-23 (Galli et al. 1998; Sikorra et al. 2016), yet the recently discovered botulinum neurotoxin-like protein encoded by Enterococcus was reported to cleave hSNAP-23, in addition to the native BoNT substrate SNAP-25 (Zhang et al. 2018). However, this study did not investigate the relative cleavage efficiency of LC/En on the substrates. To address this, we examined its activity in in vitro cleavage assays using ^35^S-labled mouse SNAP-25 and hSNAP-23. BoNT/A, BoNT/C, and BoNT/E hydrolyse peptide bonds located far from the region in which the A (embryonically expressed isoform) and B (adult isoform) SNAP-25 isoforms differ in their amino acid composition (Fig. 1A). These scissile bonds are Gln197-Arg-198, Arg-198-Ala199, and Arg180-Ile181, respectively. The amino acid difference among the SNAP-25 isoforms does not affect cleavage by BoNT/A or BoNT/E, because only the C-terminal quarter of SNAP-25 is necessary for interaction with these LCs (Vaidyanathan et al. 1999; Breidenbach and Brunger 2004). In contrast, the experimentally determined scissile peptide bond for BoNT/En, Lys-69-Asp-70, is located within the segment that differs between SNAP-25A and SNAP-25B (Zhang et al. 2018). Thus, both isoforms were included in the cleavage assays. SNAP-25A, SNAP-25B and hSNAP-23 were generated by in vitro transcription/translation in the presence of ^35^S-methionone and then incubated at various final concentrations of LC/En.

Quantification of the extent of cleavage showed that SNAP-25B is a slightly better substrate than SNAP-25A with EC_50_ values of approximately 10 to 20 nM (Fig. 1B). The estimated EC_50_ value for LC/En's activity on hSNAP-23A is approximately 500 nM (Fig. 1B), which is at least 25 times higher than the values for SNAP-25. Conversely, LC/En far surpasses the activity of LC/A in its activity on hSNAP-23. LC/A, when applied at a final concentration of 3 µM, shows ~ 7% cleavage under identical experimental conditions (Sikorra et al. 2016), suggesting an EC_50_ value of approximately 50 µM. Furthermore, the hSNAP-23 fragments generated by LC/En in our cleavage assays (Fig. S1) are compatible with the determined cleavage site for LC/En and exclude a potential second scissile peptide bond in the N-terminal cleavage product, which could not be clarified in the study by Zhang and colleagues (Zhang et al. 2018).

Overall, these data show that SNAP-25A and B are cleaved at similar rates by LC/En despite amino acids differences within the cleavage site. LC/En proteolyses hSNAP-23 at a significantly lower rate than SNAP-25, yet it is much more efficient with hSNAP-23 than LC/A. Therefore, it may be a more suitable starting basis for engineering an efficient hSNAP-23 cleaving pharmaceutical.

### Determination of the minimal substrate requirements of LC/En

As a first step to clarify the reason for LC/En's decreased sensitivity to hSNAP-23, we determined the minimal substrate requirements for substrate cleavage using SNAP-25. Three C-terminal deletion mutants were generated as fusion proteins with an N-terminal GST and a C-terminal His tag. The shortest deletion mutant, SNAP-25B-1–76, exhibited a reduced cleavability compared to the wild-type SNAP-25B fusion protein (Fig. 1C). Interestingly, the two longer C-terminal deletions mutants, 1–83 and 1–93, were less susceptible to cleavage. This indicates that SNAP-25 residues following Lys-76 may interfere with proper substrate-enzyme interaction. Apparently, longer substrate extensions C-terminal to the scissile peptide bond are not required. This is consistent with data on SNAP-25 cleavage by BoNTs, which indicate optimal cleavage of substrates with five amino acids following the scissile peptide bond (Vaidyanathan et al. 1999). Next, we analysed N-terminally truncated versions of C-terminally His6-tagged SNAP-25A for cleavability. Deletion of up to 45 amino acids at the N-terminus did not affect SNAP-25A cleavability, demonstrating that 24 amino acids at most, N-terminal to the scissile peptide bond, guarantee optimal cleavage (Fig. 1D).

### Identification of LC/En-interacting substrate residues

Next, we performed alanine scanning mutagenesis on hSNAP-23 to study how individual substrate amino acid side groups contribute to the interaction with LC/En. We focused on the area previously determined to represent the minimal substrate for efficient cleavage. Of the 26 amino acids spanning this area, nine mutations negatively affected cleavability, particularly Glu-47, Glu-50, Asn-63, Asp-65 and Met-66 (Fig. 2B). This indicates the involvement of the side chains of these residues in substrate-enzyme interactions. It was not surprising that mutation of Asn-63 and Asp-65 caused a very strong reduction in cleavability as these residues constitute the P_2_ and P_1_' positions of the cleavage site (see discussion). Conversely, mutations in three positions increased the cleavability of hSNAP-23. Substituting Met-44 or Lys-64 with alanine led to a minor increase. However, substituting Lys-49 with glycine, the amino acid present in the corresponding position of SNAP-25A and SNAP-25B (Fig. 2A), increased cleavability twofold. This suggests that the side chains of authentic hSNAP-23 residues interfere with optimal positioning at the enzyme. Of the eleven mutations to valine and one to isoleucine, four reduced the cleavability by 20% to 40% (Fig. 2C). Replacing hSNAP-23 Thr-43 with valine reduced cleavability by half. The mutant hSNAP-23-N63V was expectably largely resistant. These effects presumably indicate that the bulky valine side-chains prohibits optimal placement at the respective enzyme site. Most interestingly, replacing Asn-53 with amino acids that have acidic side chains greatly increased cleavability. Substitution with aspartic acid, the residue in the corresponding position of SNAP-25A, caused a more than threefold improvement. Substitution with glutamic acid, the residue in the corresponding position of SNAP-25B, increased cleavability by more than a factor of four.

Together, the systematic mutational analysis of hSNAP-23 and protease assays using the resulting mutants revealed the hSNAP-23 amino acid positions that mediate the interaction with LC/En. Furthermore, the data largely explain the more efficient cleavage of SNAP-25 versus hSNAP-23.

### Characterization of the catalytic mechanism of LC/En

The amino acids of BoNT LCs that mediate the peptide bond hydrolysis in their substrates are strictly conserved (Fig. 3A). LC/A His-223, His-227, and Glu-262 coordinate the catalytic Zn^2+^ together with a water molecule that is held in place by Glu-224. The arrangement of these four residues is highly similar in LC/En (Fig. 3B). Additionally, the counterparts of LC/A Arg-363 and Tyr-366 are located in corresponding positions in LC/En. LC/En residues Arg-364 and Tyr-367 are supposed to stabilize the transition state of a catalytic intermediate by forming hydrogen bond interactions with the carbonyl oxygen atoms of the P_1_' and P_1_ amino acids, respectively (Binz et al. 2002; Kumaran et al. 2008). To confirm their importance in LC/En, we analysed the activity of the mutants R364A and R364A/Y367F using SNAP-25B as the substrate. Mutating Arg-364 to alanine caused a very severe reduction in catalytic activity. Even a thousand-fold higher LC concentration (10 µM versus 10 nM) did not reach the extent of SNAP-25 cleavage measured for wild-type LC/En. The double mutant R364A/Y367F exhibited an additional strong reduction in activity. Its activity approached the detection limit (Fig. 3C). Thus, the highly similar arrangement of the key active site residues and the effect of mutating two transition state stabilizing amino acids suggests that BoNT/En's catalytic mechanism is the same as that of BoNTs.

Identification of LC/En-interacting substrate residues in hSNAP-23 revealed that mutation of the P_2_ amino acid Asn-63 and of the P_1_' amino acid Asp-65 are of paramount importance for cleavability. Accordingly, mutation of their positional counterparts in SNAP-25 should dramatically affect cleavability as well. Additionally, we also studied the effect mutating the hSNAP-23 counterpart of the P_1_ position, which is a lysine residue. Consistent with the data for hSNAP-23, mutating Asn-68 to alanine and Asp-70 to alanine or valine resulted in reductions of SNAP-25B cleavage of approximately 30- and -100-fold, respectively. Conversely, mutating Lys-69 to alanine or glutamic acid increased cleavability, similar to the effect of the mutating Lys-64 in hSNAP-23.

## Discussion

BoNT/A and BoNT/B have been used for decades as effective pharmaceuticals for treating diseases involving excessive neurotransmitter release. Recent research activities have aimed to adapt the enzymatic activity of BoNTs to different intracellular targets in order to expand their range of application to other diseases (Sikorra, al. 2016, Sikorra et al. 2020; Blum et al. 2021; Dyer et al. 2022). This study aimed to assess whether the LC of the BoNT-like protein from Enterococcus faecium could be used to develop a novel biopharmaceutical.

LC/En appears to be a suitable starting point for generating an enzyme that efficiently cleaves hSNAP-23, as it readily proteolyses this SNARE, whereas it is largely resistant to LC/A. However, in vitro cleavage assays showed that LC/En is still almost 1000-fold less efficient at cleaving hSNAP-23 than LC/A on its natural substrate, SNAP-25. This represents the biochemical basis for the successful use of BoNT/A1 as a pharmaceutical. Thus, this LC/A activity represents the benchmark. Previous studies have aimed to re-engineer LC/A1 and LC/E1, another BoNT LC with SNAP-25 as authentic substrate, for improved hSNAP-23 cleavage. In the case of LC/E, the S_2_ pocket residue Lys-224 of LC/E1 was replaced with aspartic acid to prevent repulsion of the Lys-185 substrate residue in the P_2_ position. The resulting LC/E mutant proteolyzed hSNAP-23 with approximately 10% efficiency compared to wild-type LC/E1 and the authentic substrate SNAP-25 (Chen and Barbieri 2009). However, a drawback of the LC/E approach is its much shorter duration of action compared to LC/A (Davletov et al. 2005; Tsai et al. 2010). Improving hSNAP-23 cleavage was also attempted for the significantly longer-acting LC/A1. The application of a screening method and a structure-based rational design yielded a quadruple mutant exhibiting ~ 2,000-fold increased activity towards hSNAP-23. Nevertheless, this mutant was ~ 60-fold less efficient than wild-type LC/A1 in cleaving SNAP-25 (Sikorra et al. 2020). A follow-up study applying directed evolution to this quadruple mutant resulted in an octuple mutant, leading to a further increase in catalytic efficiency (Dyer et al. 2022). Therefore, to outperform the quadruple and octuple LC/A mutants, it will be necessary to increase the hSNAP-23-directed activity of LC/En by at least a factor of 200. Availability of structure data about SNAP-25- or preferably hSNAP-23-bound LC/En would be instrumental to this endeavour. This could be achieved using an advanced in silico tool such as AlphaFold-3. Re-engineering of LC/En could then be initiated by optimizing the binding pockets for the side chains of hSNAP-23 Lys-49 and Asn-53. Their replacement by glycine and glutamic acid, respectively, largely explains the 25-fold lower cleavage rate of hSNAP-23 versus SNAP-25. An additional increase could be achieved by establishing additional ionic or H-bond interactions.

Another aspect that requires consideration is the off-target activity of LC/En versus additional SNAREs. Data published by Zhang and colleagues identified VAMPs 1, 2, and 3 as preferred substrates and indicated that, in cell-based assays at least, SNAP-25 is also a relevant target in contrast to Syx-1 (Zhang et al. 2018). Therefore, mutations introduced in LC/En to increase its activity to hSNAP-23 should reduce concomitantly in the best case its activity towards the other SNAREs. This reduction could also be achieved through additional specific mutations that mediate exclusive interactions with VAMPs or SNAP-25. Whether the off-target cleavage ability of LC/En is critical for its intended pharmaceutical use depends on the SNARE expression profile in the destined target tissue.

If the endeavours of increasing the activity LC/En to hSNAP-23 and reduction of activity to other cleavable SNARE were ultimately successful, the next step would be to identify a suitable LC/En delivery vehicle for the intended application. Pilot experiments implied that BoNT/En does not exhibit neurotoxic activity in mice or rats, which is likely due to the absence of a high affinity receptor for the cell-binding domain (H_C_) of BoNT/En. However, substituting H_C_/En with H_C_/A showed that the translocation domain (H_N_) of BoNT/En is functional with a foreign cell-binding domain (Zhang et al. 2018). A different cell-binding module must be used for LC/En, as the variant that efficiently cleaves hSNAP-23 must be delivered to the cytosol of non-neuronal tissue. Techniques that have been stablished to generate such vehicles for delivering the BoNT enzymatic domain to cells that are not naturally targeted by the toxins include the targeted secretion inhibitor (TSI) platform and the SNARE tagging technology (Darios et al. 2010; Fonfria et al. 2018). Both of these techniques are based on engineered proteins that link the LC and H_N_ domains (called LH_N_) with a binding domain that binds to a cell surface receptor on the desired target cell. After attachment to membrane receptors or cell surface proteins, which are then internalized into the cell, the H_N_ domain ensures the entry of the LC into the cytosol from a suitable endosomal compartment. In TSIs, the LH_N_ and the binding domain are directly linked, whereas in SNARE tagging, they are connected non-covalently via attached SNARE domains that form a highly stable complex. Examples of successfully produced TSIs are LH_N_/C and LH_N_/D, which are linked to epidermal growth factor and growth hormone-releasing hormone, respectively. These have been effective in decreasing the release of mucin in a pulmonary cell line and growth hormone in anterior pituitary somatotroph cells, respectively (Foster et al. 2006; Somm et al. 2012). The successful reduction of hormone secretion from various neuroendocrine tumour cell lines has been reported for LH_N_/A SNARE-tagged to ciliary neurotrophic factor, corticotropin-releasing hormone and epidermal growth factor (Arsenault et al. 2013).

Any LC/En variant with sufficient proteolytic activity against hSNAP-23 must be tested with respect to several biochemical characteristics before proceeding to the next stage of development. For example, it must be verified that none of the assembled mutations interferes with the H_N_ domain-mediated LC translocation into the cytosol. Secondly, any mutation-related effects on the structural stability must be ruled out. Additionally, potential effects on activity duration must be considered, although a comparison of the LC/En longevity versus LC/A and/or other BoNT LCs has yet to be analysed.

LC/En essentially appears to be a bona fide member of the BoNT family of zinc proteases, thereby contributing to the BoNT toolbox for developing tools for cell biology and potentially novel pharmaceuticals. This is based on the finding that it adopts a highly similar overall structure (Gregory et al. 2023), that the critical amino acids of the active centre are arranged correspondingly, and that mutations of these residues lead to reductions in catalytic activity similar to those seen with BoNT LCs. The BoNT/En LC exhibits unique characteristics in terms of the importance of hSNAP-23 substrate residues for cleavability around the scissile peptide bond. In this respect, LC/En behaves most similarly to the LC of the chimeric BoNT/HA and BoNT/F, for which the amino acids in the P2, P1' and P2' positions are highly important (Chen and Wan 2011; Lam et al. 2018). Unlike all BoNT LCs, only two additional positions in the substrate appear to be important for LC/En. These are located away from the scissile bond. The small number of interacting substrate residues may partly explain the relatively low activity towards hSNAP-23 in vitro (approximately 500 nM), compared to LC/A and LC/E, for example, which exhibit EC_50_ values at doses below 1 nM (Vaidyanathan et al. 1999).

In conclusion, the proteolytic activity of wild-type LC/En is probably insufficient to serve as a building block in the development of a novel biopharmaceutical. Thus, an accurate structural model of LC/En bound to SNAP-25- or hSNAP-23 will be essential for engineering a LC/En with optimised proteolytic activity towards hSNAP-23. The next steps could then be taken, including combining it with a suitable target tissue delivery unit and conducting assays with the final construction to demonstrate the effect of LC/En on exocytosis.

## Supplementary Information

Below is the link to the electronic supplementary material.

Supplementary Material 1

## Data Availability

No datasets were generated or analysed during the current study.

## Abbreviations

BoNT: Botulinum neurotoxin
hSNAP-23: Human 23kDA isoform of SNAP-25
LC: Light chain
SNAP-25: Synaptosomal-associated protein of 25 kDa
SNARE: Soluble N-ethyl maleimide sensitive factor attachment protein receptor
