# Supplementary material for: Characterisation of the SNAP-25 and SNAP-23 cleavage properties of the botulinum neurotoxin-like protein from Enterococcus
Source: J Neural Transm (Vienna). 2025 Dec 22;133(6):923–33. doi: 10.1007/s00702-025-03082-z (PMC13331849; doi:10.1007/s00702-025-03082-z)
Supplement: Supplementary file 1 — Supplementary Material 1 [file 702_2025_3082_MOESM1_ESM.docx]

**Supplementary Material**

**
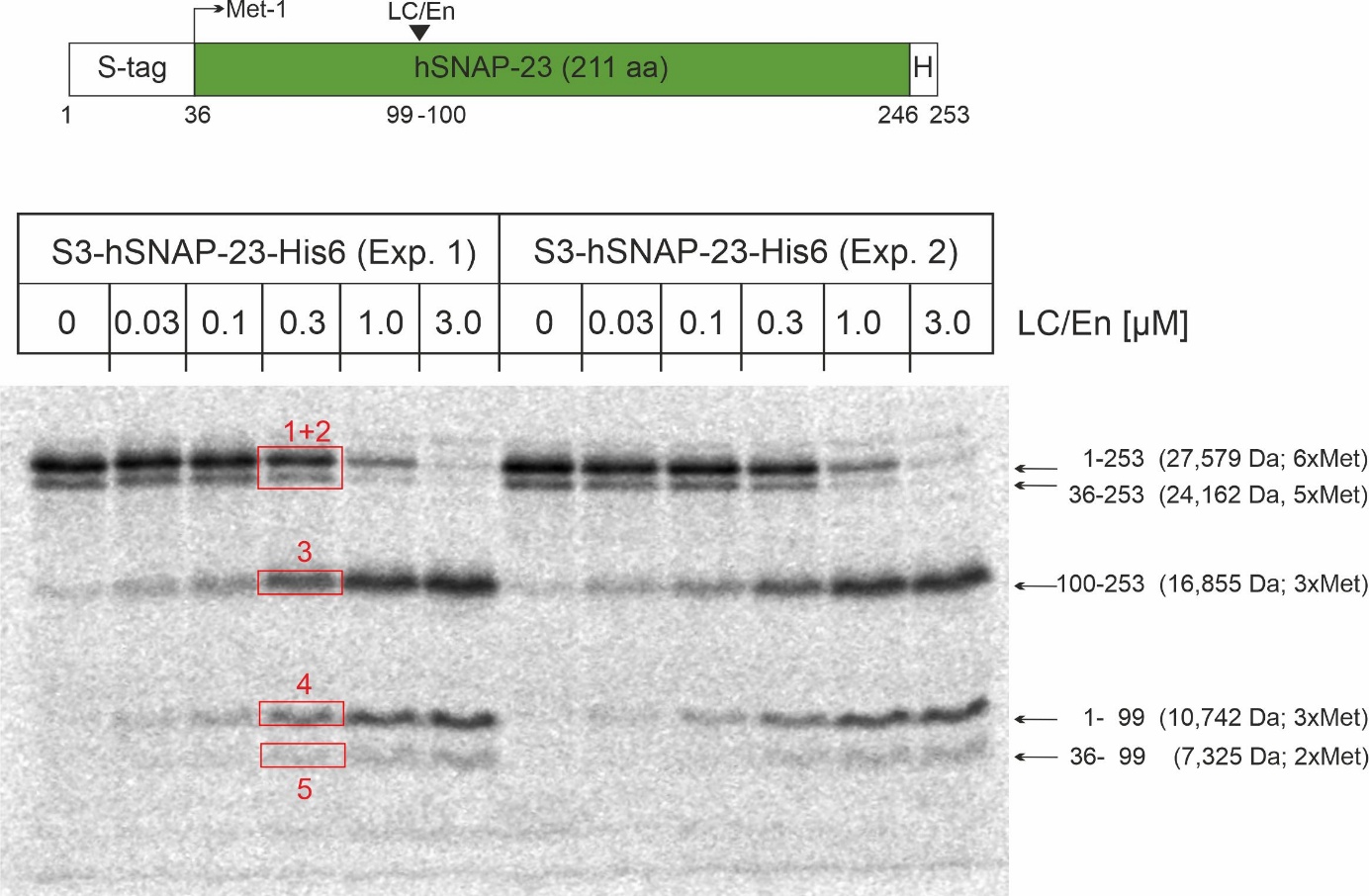
**

**Supplementary Figure 1. Cleavage of in vitro translated hSNAP-23 by LC/En.** (**top)** Schematic diagram of the hSNAP-23 fusion protein assembly used for cleavage assays with LC/En. The One-STrEP (S-tag) precedes Met-1 of hSNAP-23. A hexa-histidine-tag (H) follows the C-terminal Ser-211. The scissile peptide bond in the fusion protein, Lys-99/Asp-100, for LC/En (corresponding to amino acids Lys-64/Asp-65 of hSNAP-23) is depicted by a filled inverted triangle. (**bottom**) S3-hSNAP-23-His6 was incubated for 1 hour at 37°C with the indicated concentrations of recombinant LC/En as purified from E. coli. Reactions were stopped by the addition of sample buffer. Samples were boiled and run on 15% gels. The radiolabeled hSNAP-23 fusion protein and the generated cleavage products were visualized by phosphorimaging. The percentage of cleavage was calculated by dividing the sum of the signal intensities of bands 3, 4, and 5 (after subtraction of the corresponding signals for the untreated sample (0 µM), indicated by red rectangles) by the sum of the intensities of bands 1 to 5 (also indicated by red rectangles) and then multiplying by 100. The molecular masses as well as the number of methionines for the full-length fusion proteins and its derivatives are shown on the right. Note, hSNAP-23-His6, which lacks the N-terminal One-STrEP-tag, is produced by translation starting at the authentic start codon of hSNAP-23. This derivative is cleaved with the same efficiency as the full-length fusion protein.
